# Ultra-processed food consumption is related to screen time among Brazilian adolescents, adults and older adults

**DOI:** 10.1017/S0007114524002848

**Published:** 2025-01-14

**Authors:** Caroline dos Santos Costa, Andrea Wendt, Adriana Kramer Fiala Machado, Luiza Isnardi Cardoso Ricardo, André de Oliveira Werneck, Maria Laura da Costa Louzada

**Affiliations:** 1 Postgraduate Program in Epidemiology, Federal University of Pelotas, Pelotas, Brazil; 2 Graduate Program in Health Technology, Pontifícia Universidade Católica do Paraná, Curitiba, Brazil; 3 MRC Epidemiology Unit, University of Cambridge, Cambridge, UK; 4 Postgraduate Program in Nutrition and Public Health, University of São Paulo, São Paulo, Brazil; 5 Department of Nutrition, University of São Paulo, São Paulo, Brazil

**Keywords:** Screen time, Ultra-processed foods, Adolescents, Adults, Older adults

## Abstract

This study investigated the association between screen time and ultra-processed food (UPF) consumption across the lifespan, using data from the 2019 Brazilian National Health Survey, a cross-sectional and population-based study. A score was used to evaluate UPF consumption, calculated by summing the positive answers to questions about the consumption of ten UPF subgroups on the previous day. Scores ≥5 represented high UPF consumption. Daily time spent engaging with television or other screens was self-reported. Crude and adjusted models were obtained through Poisson regression and results were expressed in prevalence ratios by age group. The sample included 2315 adolescents, 65 803 adults and 22 728 older adults. The prevalence of UPF scores ≥5 was higher according to increased screen time, with dose–response across all age groups and types of screen time. Adolescents, adults and older adults watching television for ≥6 h/d presented prevalence of UPF scores ≥5 1·8 (95 % CI 1·2, 2·9), 1·9 (95 % CI 1·6, 2·3) and 2·2 (95 % CI 1·4, 3·6) times higher, respectively, compared with those who did not watch television. For other screens, the prevalence of UPF scores ≥5 was 2·4 (95 % CI 1·3, 4·1) and 1·6 (95 % CI 1·4, 1·9) times higher for adolescents and adults using screens for ≥ 6 h/d, respectively, while for older adults, only screen times of 2 to < 3 and 3 to < 6 h were significantly associated with UPF scores ≥5. Screen time was associated with high consumption of UPF in all age groups. Considering these associations when planning and implementing interventions would be beneficial for public health across the lifespan.

Over the past few decades, there has been a worldwide shift towards increased consumption of ultra-processed foods (UPF) and away from traditional food patterns^(1)^. According to the NOVA classification system, UPF are industrial formulations made of many ingredients and little or no whole food. They are typically high in energy, sugar, fat and sodium and contain several cosmetic substances to enhance sensorial properties such as palatability, flavour, colour and texture^(2)^. Studies have linked higher UPF consumption to several adverse health outcomes, including obesity, type 2 diabetes, CVD, various cancers, depression and all-cause mortality^(3–6)^. Data from national surveys in Brazil show that the relative share of UPF increased from 2008–2009 to 2017–2018 and corresponded to 26·5, 19·5 and 15·1 % in adolescents, adults and older adults, respectively, in the latest survey^(7)^.

Sedentary behaviour, defined as any waking behaviour with an energy expenditure of 1·5 metabolic equivalents or less while sitting or reclining^(8)^, has also increased over time and is associated with several negative health outcomes^(9,10)^. The literature indicates a relationship between sedentary behaviour and poor dietary patterns over the lifespan, although there is less consistent evidence in adults than in adolescents^(11,12)^. Some studies have found an association between television (TV) viewing and unhealthy dietary habits in adults, such as higher consumption of snacks and lower consumption of fruits, while others have found an association in the opposite direction for different types of leisure-time sedentary behaviour (e.g. computer use associated with healthy dietary habits)^(11–14)^. Moreover, there is a gap in the literature regarding the relationship between sedentary behaviour and UPF consumption as an indicator of diet quality, particularly in adults and older adults.

A previous study in Brazil, based on data from the National Survey of School Health, reported a positive association between higher leisure-time sedentary behaviour, specifically sitting time, and increased consumption of UPF among adolescents^(15)^. However, it remains unclear whether this relationship also exists for different types of sedentary behaviour and across age groups, including adults and older adults. To address this knowledge gap, our study aims to investigate the association between screen time in leisure time and UPF consumption among Brazilian adolescents, adults and older adults, considering both TV viewing and the use of computer, tablet or cell phone as separate exposures. A secondary aim is to describe the prevalence of screen time in leisure time and UPF consumption in this population.

## Methods

### Study design and sampling

Data from the second edition of the Brazilian National Health Survey (*Pesquisa Nacional de Saúde* or PNS) was used in this study. PNS is a population-based survey conducted by the Brazilian Institute of Geography and Statistics (*Instituto Brasileiro de Geografia e Estatística* or IBGE), and its sample represents the national territory and the population resident in private households in the country. The survey aims to evaluate and monitor the living and health conditions of the Brazilian population and provide relevant information to the formulation and impact evaluation of public policies^(16)^.

A main sample, from which it is possible to generate subsamples that are used in several other national surveys conducted by IBGE, was used to obtain the PNS sample. The sampling strategy was performed in three stages: from the main sample, primary sample units, composed of the census sectors or set of sectors, were selected with probability proportional to size, defined by the number of permanent private households. Then, a simple random sampling was applied to select the households from each primary sample unit selected in the first stage. The third stage comprised the simple random selection of one resident aged 15 years or over from each household to be responsible for answering the questionnaire^(16)^.

### Data collection

The questionnaire consisted of three main sections, one including questions about the household, another collecting information about all residents, with a focus on socio-economic and health characteristics, and a third section related to the selected resident. This last section included modules of questions collecting data on several topics, including lifestyles, such as diet and sedentary behaviour. Trained staff used mobile devices (smartphones) programmed with the survey questionnaire to perform the interviews in the households from August 2019 to March 2020. The 2019 edition of PNS was conducted according to the guidelines laid down in the Declaration of Helsinki, and all procedures involving human subjects were approved by the National Research Ethics Commission under decision no. 3·529·376. Informed consent was obtained from all selected residents^(16)^.

For the current purpose, we used data about TV viewing and other screen use, both expressed in hours a day and the consumption of UPF on the day prior to the interview.

TV-viewing prevalence was estimated using the following question: ‘On average, how many hours a day do you usually watch TV?’ The prevalence of other screen use was measured by the question, ‘In a day, how many hours of your free time (excluding work) do you usually use a computer, tablet or cell phone for leisure, such as using social media, watching news, videos, playing games, etc.?’ For both variables, individuals were assigned into six categories: none, less than 1 h, 1 to < 2 h, 2 to < 3 h, 3 to < 6 h, 6 h or more.

To investigate the consumption of UPF, participants were asked about the consumption (yes or no) of ten selected subgroups of UPF on the day prior to the interview, as follows: ‘Yesterday, did you drink or eat: (1) soft drink?; (2) fruit juice drink in a can or box or prepared from a powdered mix?; (3) chocolate powder drink or flavoured yogurt?; (4) packaged salty snacks or crackers?; (5) sandwich cookies or sweet biscuits or packaged cake?; (6) ice cream, chocolate, gelatine, flan or other industrialised dessert?; (7) sausage, mortadella or ham?; (8) loaf, hot dog or hamburger bun?; (9) margarine, mayonnaise, ketchup or other industrialised sauces?; and (10) instant noodles, instant powdered soup, frozen lasagne or other frozen ready-to-eat meal?’. The questionnaire was previously presented, and includes subgroups of UPF with the greatest participation in the daily energy intake estimated by the Brazilian Dietary Survey performed in the *Pesquisa de Orçamentos Familiares* (Brazilian Household Budget Survey) 2008–2009 conducted by the IBGE^(17,18)^. Using a simple sum of the positive answers given to each subgroup, it is possible to generate a score of UPF consumption that can vary from 0 to 10 points. We considered scores greater than or equal to five as the outcome, based on previous publications^(17,19)^.

Sociodemographic variables included in this study were age groups (adolescents, 15–17 years; adults, 18–59 year; older adults, 60 years and over), sex (male and female), skin colour (white, black, brown and yellow/indigenous), education level (none, incomplete elementary school, complete elementary school, complete high school and complete higher education), wealth index (in quintiles), area of residence (urban and rural) and geographic region of the country (North, Northeast, Southeast, South and Midwest). We generated the wealth index using principal component analysis including data about the number of rooms and bathrooms in the household, sewage type, assets (colour TV, refrigerator, washing machine, landline, mobile phone, microwave, computer, motorcycle, Internet access and number of cars) and existence of monthly maid/domestic employee. We categorised the wealth index into quintiles.

### Statistical analyses

First, we described the prevalence of consumption of five or more subgroups of UPF on the day before the interview (prevalence and respective 95 % CI) according to sex, skin colour, education level, wealth index, area of residence and geographic region of the country within each age group. The prevalence of TV viewing and other screen use was also described according to the age groups (adolescents, adults and older adults). Then, we presented the prevalence of consumption of five or more subgroups of UPF according to screen time within the age groups. Finally, we used Poisson regression models to assess the crude and adjusted association between screen time (TV viewing and other screen) and the consumption of five or more subgroups of UPF on the day before the interview, estimating prevalence ratios (PR) and their respective 95 % CI. Adjusted models included sex, skin colour, education level, wealth index, area of residence and geographic region of the country as potential confounders.

We performed all analyses in the Stata statistical package, version 16.1, applying the svy command, which computes standard errors by using the linearised variance estimator, and the expansion factors or sample weights. Microdata can be obtained from the IBGE website (www.ibge.gov.br).

## Results

A total of 2315 adolescents, 65 803 adults and 22 728 older adults were included in the current analyses. The overall prevalence of consumption of five or more subgroups of UPF was 28·2, 16·3 and 7·1 among adolescents, adults and older adults, respectively (Table 1). Regarding sex, the prevalence was higher for adolescent girls, while among adults and older adults, men showed a higher prevalence when compared with women. Considering skin colour, white adolescents presented the highest prevalence of UPF consumption, while for adults and older adults, the yellow/indigenous group had the highest prevalence. In terms of education, the complete elementary and high school groups showed the highest prevalence for both adults and older adults. The south region of the country presented the highest prevalence of UPF consumption, especially among adolescents. For all age groups, those living in the urban area had the highest prevalence. Regarding income, the fourth and fifth wealth index quintiles had a higher prevalence of UPF consumption. Although the prevalence of consumption of five or more subgroups of UPF was higher in the above-mentioned categories, not all of them were statistically significant based on the overlapping of 95 % CI (Table 1).

**Table 1.** Prevalence (%) and 95 % CI of scores of ultra-processed food (UPF) consumption equal to or higher than five on the day before the interview according to age group. National Health Survey, Brazil, 2019 (*n* 90 846)

	Prevalence (%) of UPF scores ≥ 5 (95 % CI)					
Variables	Adolescents		Adults		Older adults	
	(*n* 2315)		(*n* 65 803)		(*n* 22 728)	
	Prevalence (%)	95 % CI	Prevalence (%)	95 % CI	Prevalence (%)	95 % CI
Sex						
Male	27·2	23·3, 31·6	17·7	16·9, 18·6	7·6	6·7, 8·6
Female	29·3	24·8, 34·1	15·0	14·3, 15·7	6·7	6·0, 7·6
Skin colour						
White	31·8	26·3, 37·8	17·3	16·4, 18·2	8·3	7·4, 9·3
Black	26·6	18·0, 37·5	16·3	14·9, 17·7	6·6	5·0, 8·7
Brown	26·2	22·6, 30·3	15·2	14·5, 16·0	5·4	4·6, 6·4
Yellow/Indigenous	20·0	5·1, 53·5	20·9	15·3, 27·9	11·4	5·9, 21·0
Education level*						
None	–		10·6	7·4, 15·0	3·5	2·6, 4·7
Elementary incomplete	–		10·5	9·7, 11·4	6·0	5·2, 6·9
Elementary complete	–		18·8	17·5, 20·3	10·3	8·0, 13·2
High school	–		19·4	18·5, 20·4	11·4	9·4, 13·6
Superior (tertiary)	–		15·6	14·4, 16·9	8·4	6·9, 10·4
Wealth index						
1st (poorest)	15·6	11·1, 21·6	10·1	9·1, 11·1	4·1	3·3, 5·2
2nd	25·0	19·2, 31·9	13·9	12·9, 14·8	4·6	3·6, 5·9
3rd	30·6	24·2, 37·8	16·9	15·8, 18·2	7·1	5·9, 8·4
4th	38·0	30·5, 46·1	18·4	17·2, 19·7	9·0	7·5, 10·7
5th (wealthiest)	28·1	21·8, 35·5	18·2	17·0, 19·5	9·9	8·4, 11·6
Area of residence						
Urban	30·5	26·9, 34·3	17·5	16·9, 18·1	7·8	7·1, 8·5
Rural	18·0	14·0, 22·9	8·6	7·9, 9·4	3·3	2·7, 4·0
Region						
North	23·7	18·8, 29·3	13·7	12·7, 14·8	3·4	2·7, 4·3
Northeast	25·7	21·7, 30·2	10·4	9·8, 11·0	2·7	2·1, 3·5
Southeast	27·3	21·4, 34·0	18·7	17·6, 19·8	8·7	7·6, 10·0
South	41·2	32·1, 51·0	22·5	21·2, 23·8	11·5	9·9, 13·2
Midwest	27·2	19·0, 37·3	14·8	13·5, 16·1	5·7	4·6, 7·0
Total	28·2	25·2, 31·4	16·3	15·7, 16·8	7·1	6·5, 7·8

Missing values: Skin colour, *n* 10; *Education level not presented for adolescents because is related to age.

**Figure 1.**
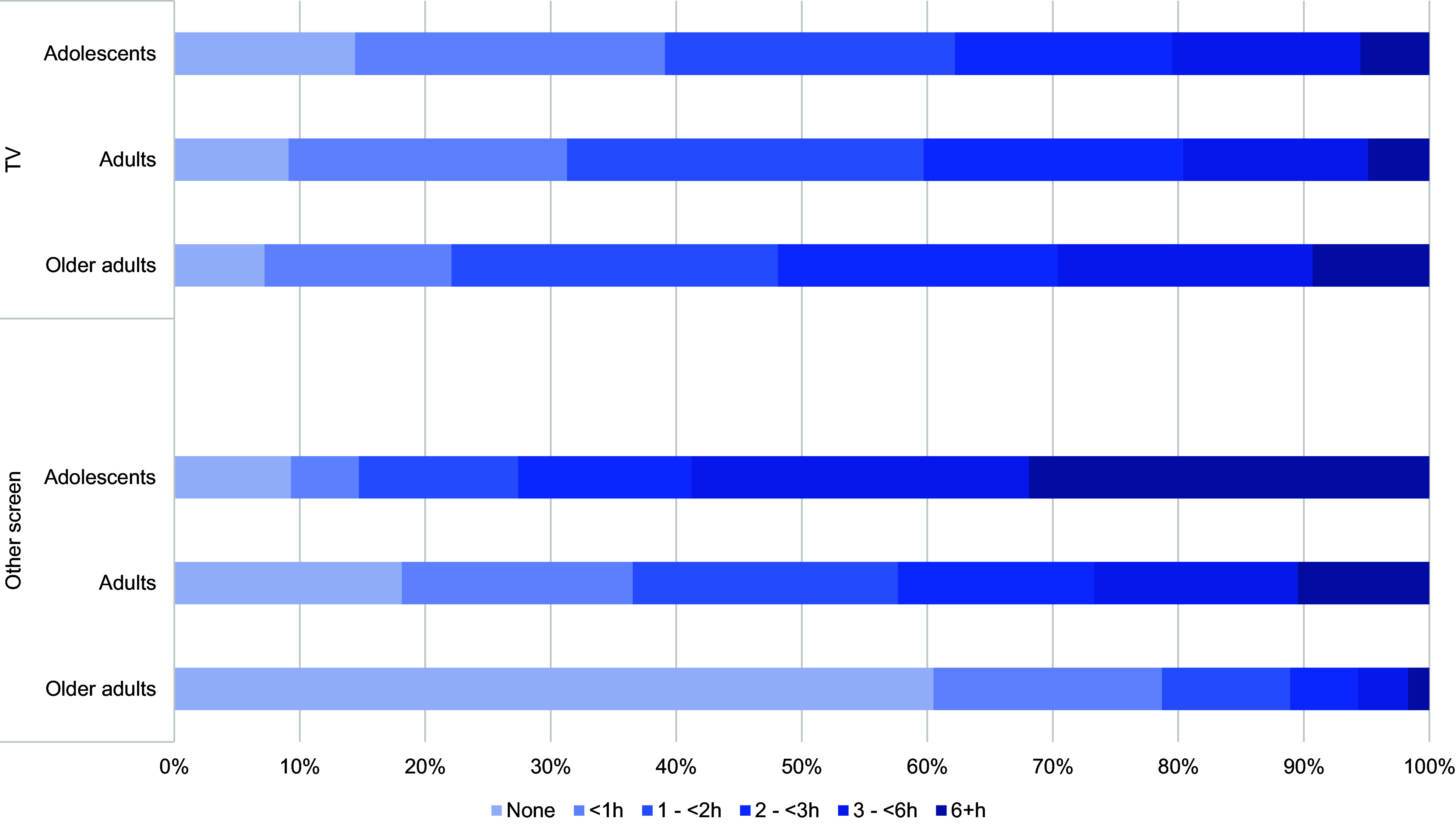
Screen time distribution according to age group. National Health Survey, Brazil, 2019 (*n* 90 846).

Figure 1 shows the screen time distribution according to age group. About 38 % of adolescents watch TV for over 2 h, and this seems to be similar for adults (around 40 %) but higher among older adults (52 %). For all age groups, less than 10 % of the sample watches TV for over 6 h. Regarding other screens, the pattern is reversed when compared with TV, with adolescents spending substantially more time using screens than adults and older adults. Over 30 % of adolescents spend more than 6 h on other screens, while for adults, this proportion is only 10 % and among older adults less than 2 %. Also, around 60 % of older adults do not engage with other screens.

**Figure 2.**
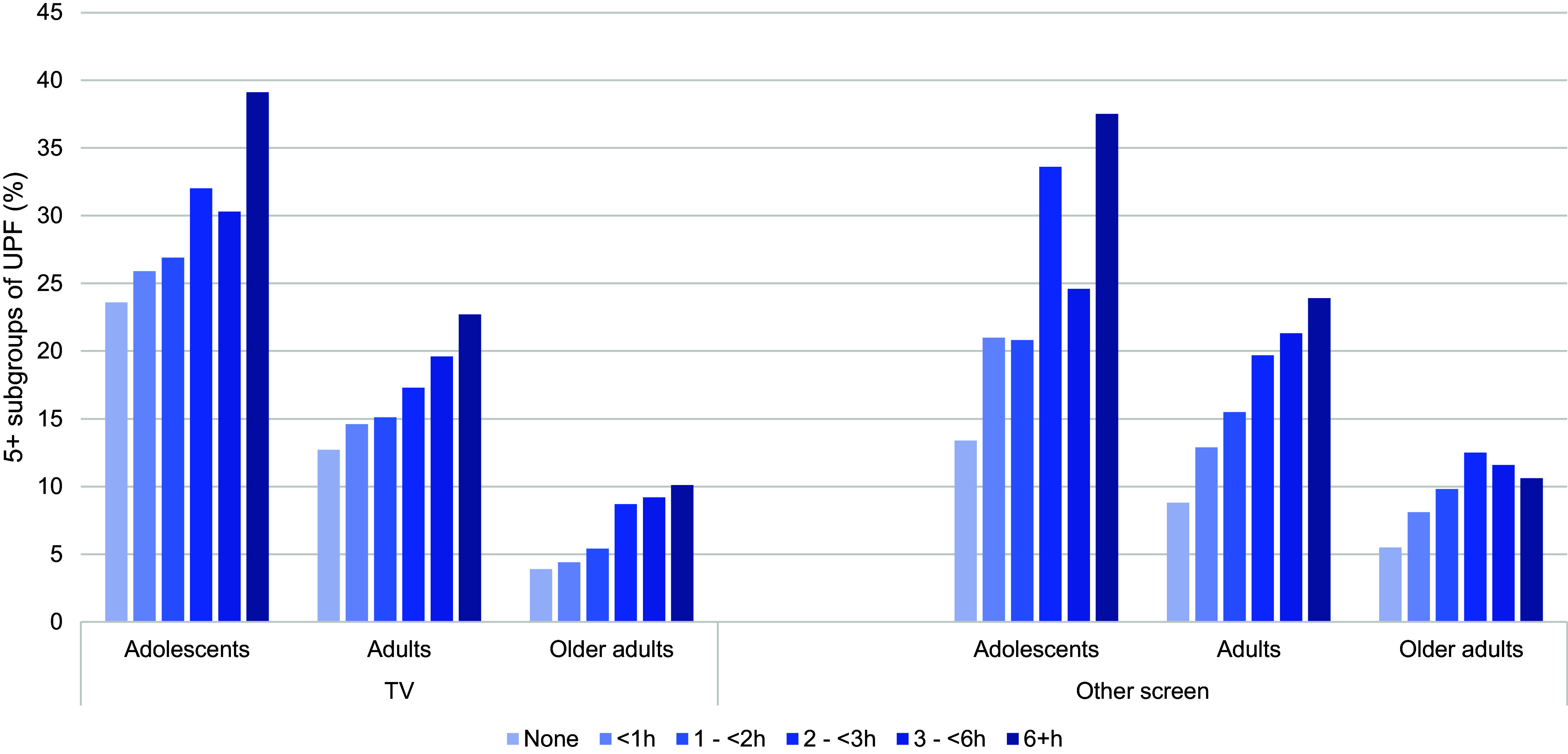
Consumption of five or more subgroups of ultra-processed foods (UPF) according to screen time and age groups. National Health Survey, Brazil, 2019 (*n* 90 846).

In general, consumption of five or more subgroups of UPF on the previous day was positively associated with TV and other screen time for all age groups (Fig. 2). For adolescents, there is a 16 percentage points difference in the prevalence of five or more UPF between no TV time and 6 or more hours of TV. Adults presented 10 percentage points of difference between the extreme TV time categories. Despite older adults having a lower prevalence of consuming five or more UPF, those who watched over 6 h of TV had a prevalence of 6·2 percentage points higher than those who did not watch TV. On the other hand, those who engaged with other screens for 6 h or more a day presented a prevalence of consuming five or more UPF on the previous day, with 24·1, 15·1 and 5·1 percentage points higher for adolescents, adults and older adults, when compared with those who did not use other screens. Furthermore, for older adults, the 2 to < 3 h of other screen time stood out with a high prevalence of five or more UPF, followed by a slight decrease in the next categories.

**Figure 3.**
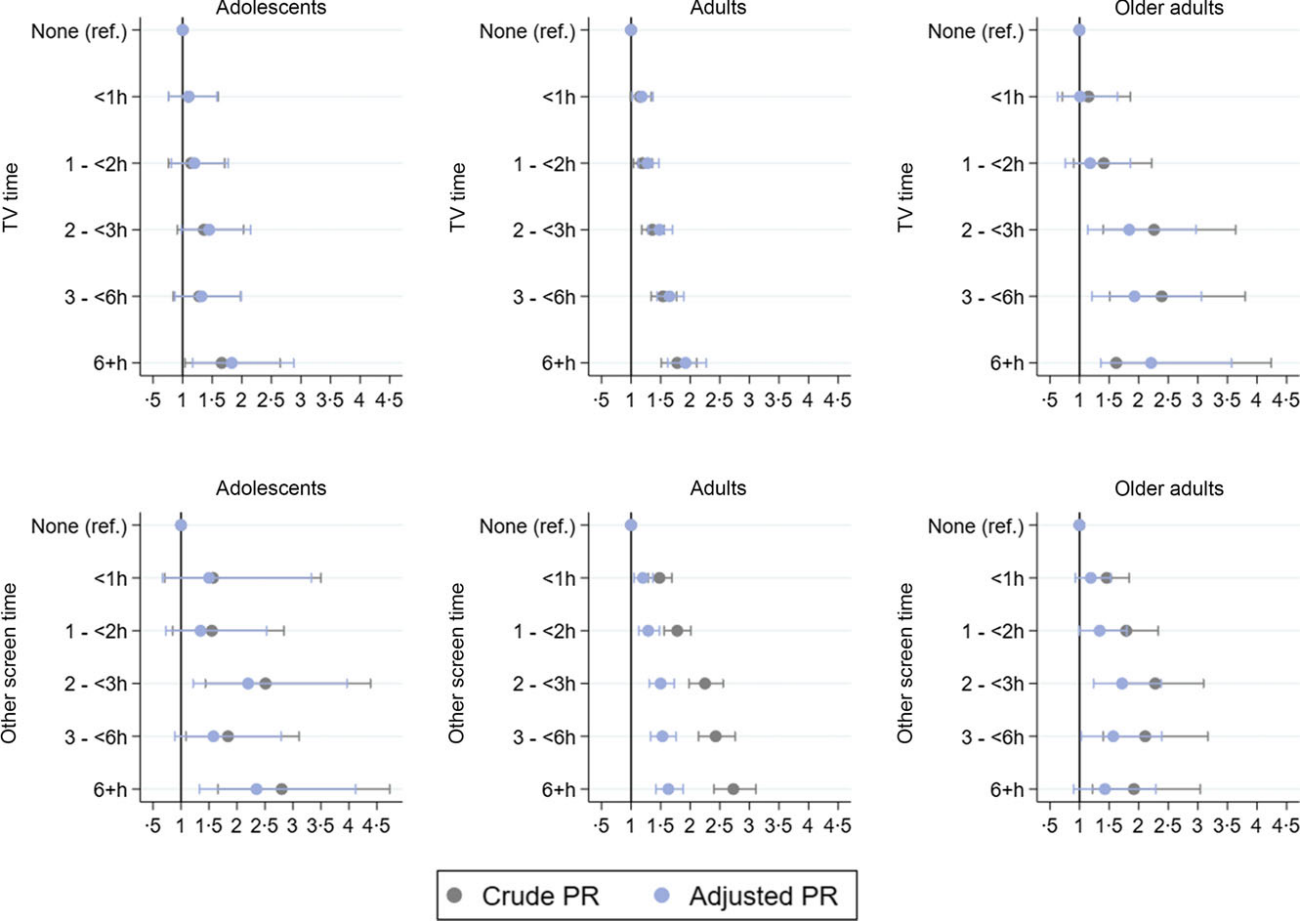
Crude (*n* 90 846) and adjusted (*n* 90 836) association between screen time and the consumption of five or more subgroups of ultra-processed foods on the day before the interview. National Health Survey, Brazil, 2019. Adjustment: sex, age, skin colour, education level, wealth quintiles, area of residence and geographic region of the country; PR, prevalence ratio.

Figure 3 presents the crude and adjusted association between screen time and UPF consumption. When adjusting to sex, age, skin colour, education level, wealth quintiles, area of residence and region, adolescents with 6 or more hours of TV time had a prevalence of 1·83 (95 % CI 1·17, 2·88) times higher of consuming five or more UPF when compared with those who do not watch TV. When observing the specific categories, significant results were only found for the highest level of TV time. Considering all the categories, a dose–response was found, with a *P*-value for linear trend of 0·006. For adults, there was a statistically significant increase in UPF consumption for all categories of TV time, with a gradual increase in the PR with the hours of TV (*P*_for linear trend_ <0·001). A similar pattern was observed for older adults but with a significant increase only from 2 to < 3 onwards (*P*_for linear trend_ <0·001).

Regarding other screens, adolescents engaging for 2 to < 3 and over 6 h showed a prevalence 2·20 and 2·35 times higher, respectively, of consuming five or more UPF in comparison to the ‘none’ category. The remaining specific categories were not statistically significant. Considering all the categories, a dose–response was found, with a *P*-value for linear trend of 0·001. Among adults, engaging for over 1 h with other screens results in a PR of 1·29 for consuming five or more UPF, increasing steadily and significantly with the increased time using other screens, up to 1·63 in the 6 or more hours category (*P*_for linear trend_ < 0·001). For older adults, significant results were only obtained for the 2 to < 3 h and 3 to < 6 h categories, which had a UPF consumption 1·72 and 1·57 times higher than the reference group, but a dose–response was found when considering all the categories (*P*_for linear trend_ < 0·001) (Fig. 3).

## Discussion

Findings from this population-based study shed light on the relationship between screen time and UPF consumption. Specifically, we found that higher screen time was generally associated with increased consumption of UPF, with a clearer dose–response pattern observed among adults and older adults, particularly when considering TV time as exposure. In contrast, when considering other screen use, the magnitude of the association seemed to be higher in adolescents than in adults or older adults. Our analyses also highlight the prevalence of screen time across different stages of life, as well as age-related differences in UPF consumption.

Our study identified a concerning prevalence of prolonged screen time in all age groups, particularly in adolescents and adults. While the WHO recommends limiting sedentary behaviour^(20)^, Canada’s 24-h movement behaviour guidelines set specific limits for recreational screen time, recommending no more than 2 h per d for children and adolescents and 3 h for adults and older adults^(21,22)^. We found that nearly four in ten adolescents exceeded the recommended limit for TV time, while approximately 20 and 30 % of adults and older adults, respectively, had more than 3 h per d of TV time. Additionally, we found that 73 % of adolescents, 27 % of adults and 6 % of older adults exceeded the recommended limit for other recreational screen time (e.g. computer, tablet or cell phone use). It is important to note that our data were collected into categories and not in continuous hours, so the actual prevalence of combined TV and other screen use above the recommended threshold may be even higher. Our findings are a call for interventions targeting to reduce the different types of sedentary behaviour across different age groups.

Adolescents presented a higher prevalence of excessive consumption of UPF when compared with their counterparts. Conversely, older adults had the lowest prevalence among the three age groups. These findings align with the national trend in Brazil, where the proportion of energy intake from UPF was 26·5 % among adolescents, 19·5 % in adults and 15·1 % in older adults, according to the latest edition of the Brazilian Household Budget Survey^(7)^. The inverse relationship between age and consumption of UPF has been observed in other countries as well and could be attributed to factors such as higher exposure to marketing of these products, especially targeting children and adolescents^(23)^; a cohort effect, where people in older age groups grew up with less availability of UPF and may have developed healthier food preferences; or a greater awareness about health and nutrition as people age^(24)^.

Regarding the relationship between screen time and consumption of five or more subgroups of UPF, we found significant associations across the three age groups regardless of whether the screen time was spent watching TV or using other devices such as computers, tablets or cell phones during leisure time. In addition to the habitual snacking while watching screens, it is possible that exposure to the advertising of UPF could contribute to this association. Previous studies have shown that eating while using screens is linked to greater consumption of UPF, even when main meals such as lunch and dinner are eaten in front of the TV^(25,26)^. Ultra-processed foods are designed to be convenient, practical and portable and are marketed as snacks or ready-to-eat meals. They can easily replace freshly prepared meals made with natural or minimally processed foods^(2)^. Moreover, UPF are often hyperpalatable and can disrupt the body’s natural hunger and satiety signals, and eating them while engaging with screens could exacerbate ‘mindless’ overconsumption of these foods^(27,28)^. A study in Brazil found that over 90 % of the foods advertised on TV and other social media are ultra-processed, and most marketing strategies used are considered persuasive, including emotional and sentimental appeals to encourage consumption^(29)^. Finally, other studies have shown that risk factors for unhealthy behaviours, such as insufficient physical activity and unhealthy eating, tend to co-occur and are not independently distributed in the population^(30,31)^.

Although screen time has presented an association with a higher consumption of UPF at different stages of life and types of screens, the patterns of this relationship seem to differ across subgroups. For TV viewing specifically, while a clearer dose–response from the first category of TV hours onwards and excessive consumption of UPF increase was found for adults, among adolescents and older adults, this was observed only for 6 h or more and from 2 h onwards, respectively. This result is not in line with another study with data from Brazilian adolescents, which described a dose–response association between the use of screens and consumption of UPF^(15)^. In the present study, the PR for 6 h or more was similar across the age groups.

When considering other screen use as exposure, the PR seems to present a higher magnitude in adolescents than in their counterparts. The prevalence of excessive UPF consumption was 140 and 60 % higher for those adolescents and adults engaging with other screens for over 6 h a day, respectively. Variations in the content to which adults and adolescents engage may impact their consumption of UPF differently. Adolescents may be more exposed to non-regulated advertisements for UPF on social media and gaming apps, which could lead to increased consumption of these products. A previous study showed that, among a sample of YouTube videos promoted by the most popular kid influencers (ages 3–14 years) in 2019, 43 % of the videos featured food, 90 % of which were unhealthy branded products^(32)^. Experiences with advertisements may have the power of shaping food brand preferences of children and adolescents, mainly when they are connected to prizes or collectible gifts or when they dialogue directly with this population subgroup^(29,33)^. In contrast, adults could spend more time engaging in other hobbies or interests, such as reading books or watching movies besides social media and may be less exposed to such advertisements. Although children and adolescents are the most vulnerable, persuasive marketing content can influence individuals of all ages, explaining our dose–response findings for adults in both TV and other screen use^(23,34)^. Policymakers should consider these peculiarities related to age on the relationship between screen time and food choices when planning strategies and actions.

In relation to UPF, although Brazil has implemented some regulations and policies aimed at controlling its consumption, significant challenges remain in several areas. The Strategic Action Plan for Tackling Chronic Noncommunicable Diseases recognises UPF as risk factors, and initiatives such as the update of the National School Feeding Program and the new nutritional labelling regulations from 2020 represent important progress. However, the country has yet to adopt more robust price regulation measures, such as selective taxation of these products, despite evidence of their effectiveness in controlling obesity rates. Additionally, the regulation of advertising, especially targeted at children, lacks more concrete enforcement. While legislation recognises advertising directed at children as abusive, specific regulations to ensure its effective implementation are still missing.

This study presents both strengths and limitations, which should be taken into consideration when interpreting its results. Although our hypotheses are mostly focused on the possible role of screen time on UPF consumption, we are aware that the cross-sectional design prevents making directional or causality conclusions, which means it is not possible to determine whether screen time causes greater consumption of UPF or if it represents an effect of the latter. Nevertheless, both screen time and UPF consumption are unhealthy behaviours that require attention in public health policies as they increase the risk of non-communicable diseases. The smaller sample size among adolescents and older adults could lead to a lack of statistical power, and conclusions about these age groups should be made with caution. Furthermore, associations found for intermediate but not extreme categories of other screen time in these two groups could possibly be explained by residual confounding. Self-reported information on both UPF consumption and screen time can be prone to desirability and recall biases or an underreporting of food consumption can occur, mainly among older adults^(35)^. Additionally, food consumption was not assessed using a more detailed instrument such as a 24-h dietary recall, not accounting for quantities and assuming equivalency across items, or an appropriate tool to estimate frequency and usual consumption, as the FFQ, which can lead to a biased classification. However, the questionnaire used to generate the scores of UPF consumption is simple and easy to understand when compared with more complex instruments. Also, a performance study showed that a similar score for UPF consumption has good potential in reflecting the dietary share of UPF when compared with a tool that considers quantities^(19)^. The score of UPF consumption was previously presented in the PNS sample and has been identified as an important tool for evaluating and monitoring the consumption of these products in surveillance systems, such as national population-based studies^(17)^. The representativeness of a population-based study at national and regional levels, including adolescents, adults and older adults, is noteworthy. Finally, it was not possible to differentiate the ‘other screen’ devices since the questionnaire asked all the devices together. It would be relevant to explore which device has more impact on the consumption of UPF. However, evaluating the time engaging with other screens separated from TV time allowed us to show the association of UPF consumption with two types of sedentary behaviour, whose prevalence differs across the lifespan, highlighting the high prevalence of older adults engaging more with TV and adolescents with cell phones, computers and tablets in their leisure time.

Our study provides evidence of a clear association between screen time and higher consumption of UPF in individuals across different age groups, including adolescents, adults and older adults. These findings suggest that public policies aimed at reducing screen time could have multiple benefits, not only improving overall health and well-being by increasing physical activity levels but also contributing to a reduction in UPF consumption. Additionally, it is crucial to consider regulating the advertising of UPF in the media, particularly those targeted towards children and adolescents, to further reduce the negative impacts of screen time and promote healthy eating habits.
